# Middle mediastinal paraganglioma enclosing the left anterior descending artery: A case report

**DOI:** 10.1097/MD.0000000000030377

**Published:** 2022-09-02

**Authors:** Bing Zhang, Guofang Liu, Jian Li, Pinghua Wan, MD

**Affiliations:** a Department of Radiology, The Chenjiaqiao Hospital of Shapingba District of Chongqing, China; b Department of Radiology, Second Affiliated Hospital, Army Medical University, Chongqing, China; c Department of Cardiovascular surgery, Second Affiliated Hospital, Army Medical University, Chongqing, China.

**Keywords:** case report, computed tomography, coronary artery bypass, mediastinal tumor, neuroendocrine tumor

## Abstract

Paragangliomas are extremely rare neuroendocrine tumors arising from ganglia. These tumors are barely found in the middle mediastinum.

**Patient Concerns:**

A 31-year-old male patient was admitted to our hospital for treatment of an middle mediastinal tumor which was found incidentally on echocardiography during a medical checkup. Contrast-enhanced chest computed tomography (CT) demonstrated a well-defined hypervascularized heterogeneous mass located in the middle mediastinum. The tumor showed strong 18F-fluorodeoxyglucose (FDG) activity on positron emission tomography-computed tomography (PET-CT).

**Diagnosis:**

Before the surgery, we presumed the mass was an angiogenic or neurogenic tumor. As a result, the histological features favored a diagnosis of paraganglioma.

**Interventions:**

The tumor was completely removed by anterior thoracotomy, along with the proximal segment of the left anterior descending artery (LAD), and coronary artery bypass grafting (CABG) was performed immediately after the tumor excision.

**Outcomes:**

The patient had an uneventful recovery. The patient did well in the postoperative follow-up without any complications and signs of recurrence at 3 months, 1 year, 2 year and 4 year.

**Lessons:**

This report can increase the confidence in surgeries of mediastinal paragangliomas adhering tightly the adjacent structures.

## 1. Introduction

Mediastinal paraganglioma is an extremely rare tumor derived from extraadrenal chromaffin cells, which accounts for no more than 0.3% of all mediastinal tumors and <2% of all paragangliomas.^[1,2]^ Mediastinal paraganglioma can be either functional with the ability to synthesize and release catecholamine or nonfunctional without the catecholamine secretion. Clinical symptoms may be caused by catecholamine excess (headache, tachycardia and sweating) or due to the mass effect (dyspnea, dysphagia and chest pain).^[3]^ However, most patients are asymptomatic and discovered occasionally.^[4]^ Mediastinal paragangliomas are highly vascular, which often adhere firmly to the adjacent mediastinal structures, such as the great vessels, heart, trachea and spine.^[5,6]^ Here we present the first case of a nonfunctional middle mediastinal paraganglioma, which encloses the left anterior descending artery (LAD). The tumor was completely removed by anterior thoracotomy, along with the proximal segment of the LAD, and coronary artery bypass grafting (CABG) was performed immediately after the tumor excision.

## 2. Case report

**Figure 1.**
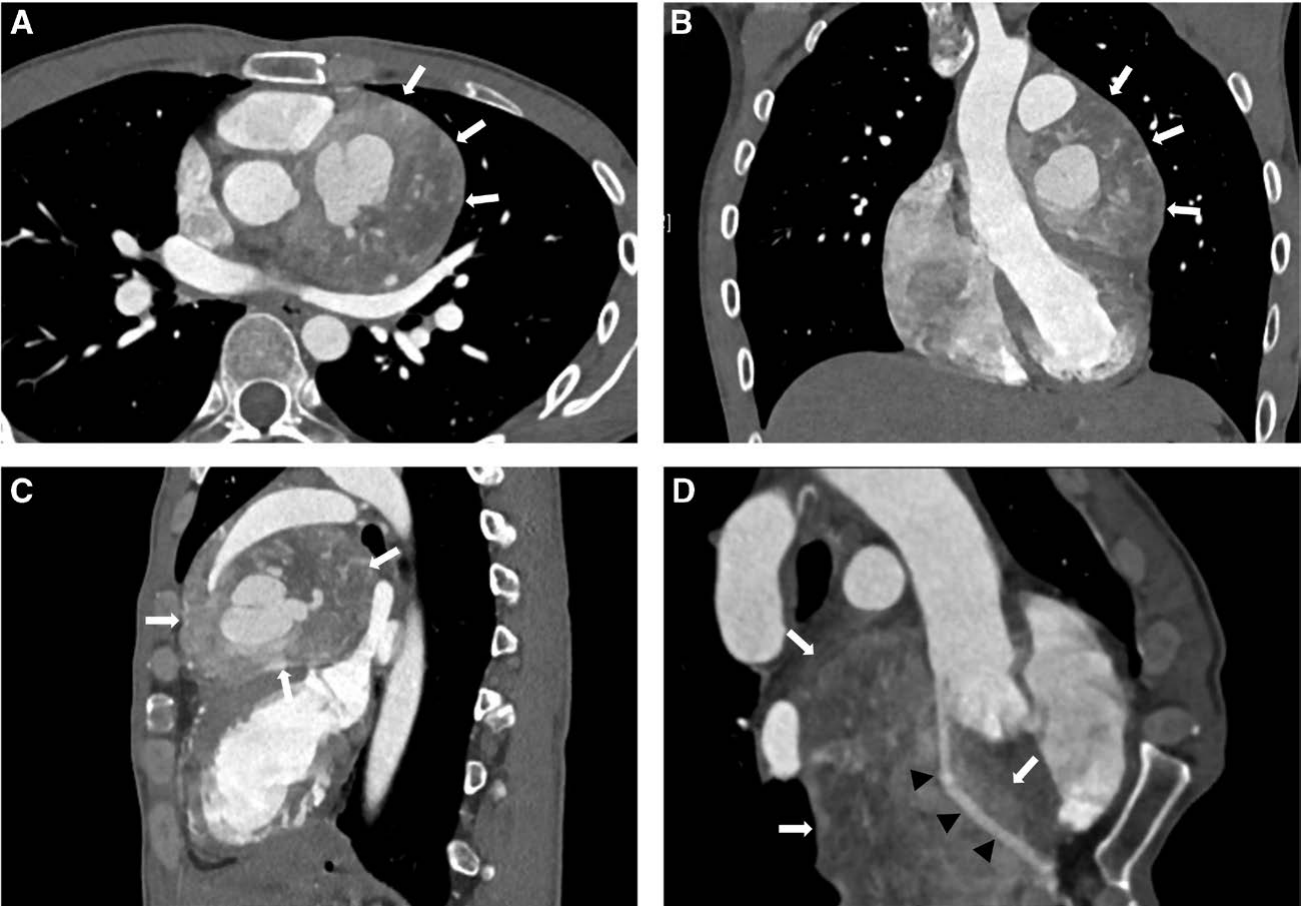
Axial (A), coronal (B) and sagittal (C) contrast-enhanced computed tomography images showed a hypervascularized heterogenous mass in the middle mediastinum (arrows). A magnified multiplanar curved reformat view of the proximal left anterior descending artery (arrowheads) showed that it was enclosed by the tumor (arrows), but the lumen was patent (D).

**Figure 2.**
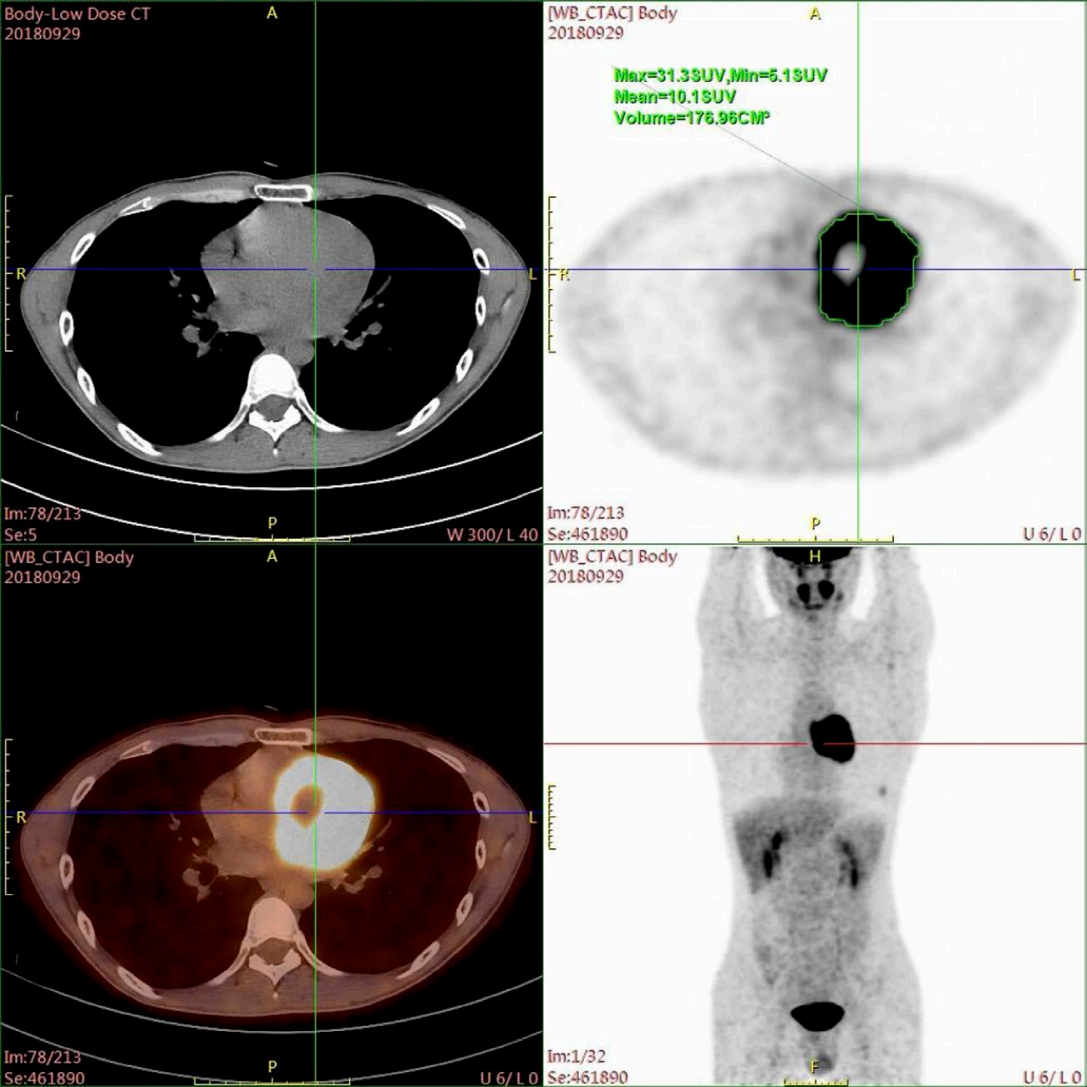
Positron emission tomography-computed tomographic scan demonstrated a marked accumulation of ^18^F- fluorodeoxyglucose in middle mediastinal mass.

A 31-year-old male patient was admitted to our hospital for treatment of an middle mediastinal tumor which was found incidentally on echocardiography during a medical checkup. He had no history of hypertension, headache or other specific symptoms. Electrocardiogram showed flat T-waves in lead I but no abnormalities in other leads. He was found to be positive for hepatitis B virus during the laboratory examination, but other laboratory data were within normal limits. Contrast-enhanced chest computed tomography (CT) demonstrated a well-defined hypervascularized heterogeneous mass measuring 7.7 × 7.2 × 6.3 cm located in the middle mediastinum (Fig. 1A–C), which followed a rapid peak and washout enhancement pattern. The tumor was adherent to the heart and the multiplanar curved reformat view showed it enclosed the proximal left anterior descending artery (LAD), but the lumen of the proximal LAD was absolutely patent without stenosis (Fig. 1D). The tumor showed strong ^18^F-fluorodeoxyglucose (FDG) activity on positron emission tomography-computed tomography (PET-CT), with a maximum standardized uptake value (SUV_max_) of 31.3 (Fig. 2).

Based on the findings, we presumed the mass was an angiogenic or neurogenic tumor. The patient was scheduled for surgery due to its malignant potential. Surgical resection was going to be performed under cardiopulmonary bypass and cardiac arrest for the size and the location of the tumor. The pericardium was opened via a standard midline sternotomy. Intraoperatively, an encapsulated, highly vascularized, 8 × 8 × 7 cm mass was noted on the left side of the ascending aorta, between right ventricular outflow tract, pulmonary artery and left atrium. Several feeding vessels from aortic arch and pulmonary artery were identified and clipped. At first, the tumor was resected from the surrounding structures with relative ease. However, as the excision progressed caudally, the proximal LAD was found enclosed firmly by the tumor and cannot be separated completely. Thus, the proximal LAD was resected together with the mass and CABG was performed. The left greater saphenous vein was utilized as the coronary conduit. Perioperative blood loss was 460 ml. No obvious blood pressure fluctuations occurred during surgery. The duration of cardiopulmonary bypass and aortic cross-clamp was 297 min and 167 min respectively.

**Figure 3.**
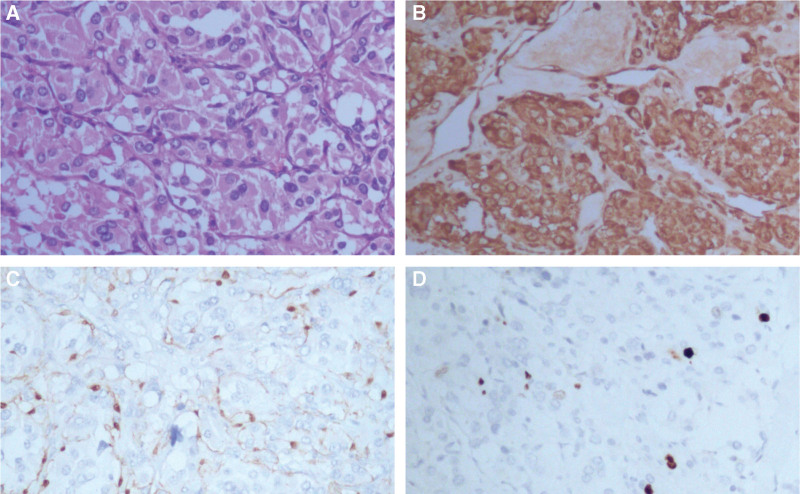
The histologic and immunohistochemical features of the tumor tissue. The tumor cells arranged in nests with vascular stroma (A). The tumor cells were positive for chromogranin (B), S-100 protein (C) and the Ki-67 index was 5% (D). Magnification, ×200.

Histologically, the tumor consisted of polygonal cells arranged compactly in nests (Zellballen) which were bordered by vascular stroma with rich fine capillaries. The cells had spindle nuclei, fine chromatin, inconspicuous nucleoli and abundant eosinophilic granular cytoplasm. Mitosis, foci of necrosis and invasion into adjacent structures were rarely present (Fig. 3A). Immunohistochemically, the tumor cells were positive for chromogranin (Fig. 3B), and S-100 protein (Fig. 3C) and had an low index of proliferation (Ki-67 index of 5%) (Fig. 3D). There were no signs of malignancy. These features favored a diagnosis of paraganglioma.

**Figure 4.**
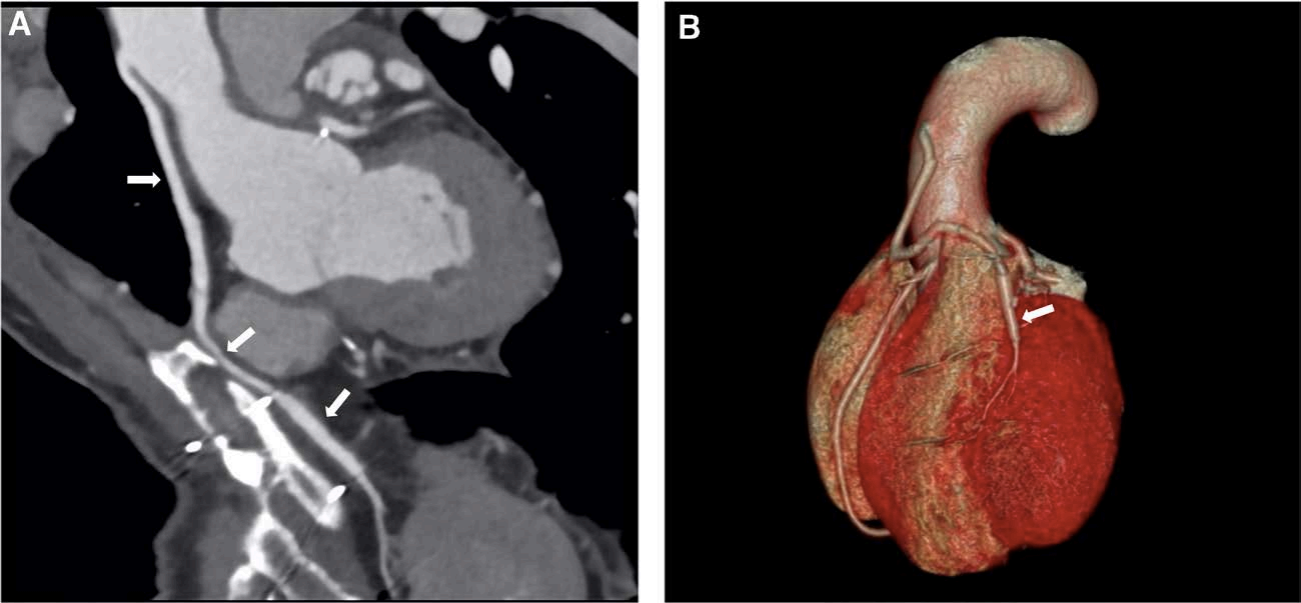
Multiplanar reformat image showed the saphenous vein graft to the left anterior descending artery was absolutely patent (A, arrows). The 3-dimensional visualization of the heart and coronary arteries revealed no disorders of the graft (B, arrows).

The patient did well in the postoperative follow-up without any complications and signs of recurrence on echocardiology and CT scans at 3 months, 1 year, 2 year and 4 year. Coronary computed tomography angiography (CCTA) was performed 4 year after surgery for evaluation of CABG patency and no stenosis or other vascular disorders with the graft was found (Fig. 4).

## 3. Discussion

Pheochromocytomas and paragangliomas are relatively rare neuroendocrine tumors which originate from chromaffin cells. The only difference between them is the anatomical location. The former is located in adrenal gland while the latter can be anywhere extraadrenal.^[7]^ Paragangliomas are frequently located in the retroperitoneum, sometimes in the pelvis, the head and the neck and seldom in the mediastinum. Mediastinal paragangliomas located in the posterior mediastinum often arise from paravertebral ganglia and tend to be functional with catecholamine secretion. The most frequent complaints of functional paragangliomas secondary to hypercatecholamine are headache, tachycardia, arrhythmia and sweating.^[8]^ Combined with clinical symptoms, elevated metanephrines in plasma and urine can strongly support the existence of those tumors. While mediastinal paragangliomas located in the anterior or middle mediastinum often arise from para-aortic ganglia and tend to be nonfunctional.^[9–11]^ Those patients with nonfunctional tumors are generally noted occasionally during imaging studies or related symptoms of compression or invasion of adjacent structures, such as dysphagia, chest pain and dyspnea.^[10]^ As in our case, the patient didn‘t show any complaints and the mass was first discovered by echocardiography during the medical check.

Imaging methods show great importance in diagnosis of mediastinal paragangliomas, which can provide the location and extension of the lesion and assess its invasiveness. Paragangliomas are usually highly vascular and demonstrate intense and heterogenous enhancement on CT and magnetic resonance imaging (MRI). Although MRI was not performed in this case, it also play an importance role in the diagnosis. The intermediate signal intensity on T1-weighted images and high signal intensity on T2-weighted images is a characteristic feature of this kind of tumor. Most paragangliomas are avid for ^18^F-FDG, which was also seen in our case. Although the highly uptake of ^18^F-FDG is nonspecific to paragangliomas, it is still of great value to determine whether there is metastasis.^[9]^

The differential diagnosis for a middle mediastinal mass includes congenital cysts, lymphadenopathy and angiosarcoma. Congenital cysts, including pericardial cyst, esophageal cyst and bronchial cyst, usually appear as well-circumscribed water like density/intensity with thin walls. They never show any enhancement on CT or MRI and thus can be excluded.^[12]^ Lymphadenopathy, like malignant lymphoma and metastasis, sometimes shows marked contrast enhancement on CT or MRI. However, metastatic tumors can be ruled out by revelation of the original lesion. Angiosarcoma usually demonstrates pronounced heterogenous contrast enhancement resembling paraganglioma, which is the most controversial differential diagnosis.^[8,10]^ Thus, histological and immunohistochemical evaluation are necessary to make a definitive diagnosis. The microscopic pattern of paragangliomas are special. The tumor cells are arranged in a typical nesting pattern and separated by abundant microvasculature. The cells have round or oval nuclei and eosinophilic cytoplasm. We can occasionally identify a few mitoses or mild to moderate nuclear pleomorphism in the proliferative tumor cells.^[13]^ Paragangliomas are intensely immunoreactive to chromogranin, synaptophysin and S-100 protein but negative for cytokeratin and epithelial membrane antigen.^[7]^

Although paraganglioma is typically benign, it still has malignant potential, including local invasiveness and distant metastasis.^[3,14]^ Surgical resection is the preferred therapeutic strategy for paraganglioma due to its resistance to chemotherapy and radiotherapy.^[2,15]^ As paraganglioma is highly vascular and adhere firmly to adjacent great vessels or heart, the operation can cause massive bleeding. Therefore, the vascular supply of the tumor should be evaluated by preoperative angiography and preventive embolization can be performed if necessary. In our case a preoperative embolization did not be performed because there were no apparent nourishing blood vessels arising from the adjacent great vessel. In addition, there was no massive bleeding during the operation. Cardiopulmonary bypass is often adopted to ensure a safe and complete excision. Sometimes the adjacent tissues can be excised together with the tumor.^[16,17]^ As in our presented case, the proximal LAD was resected and revascularized with a great saphenous vein graft. For functional paragangliomas, catecholamine crisis is another concern. Thus, adrenergic blockers and calcium channel blockers can be used preoperatively to avoid perioperative hypertensive crisis. After surgery, a regular follow-up is needed to evaluate whether there is local recurrence or metastatic spread. Fortunately, majority parents who achieved successful resection have a favorable prognosis.^[18–20]^

In conclusion, we experienced an extremely rara case of asymptomatic and nonfunctional paraganglioma arising from the middle mediastinum, which firmly enclosed the proximal LAD. To ensure a complete resection of the tumor, a portion of the LAD was excised and revascularized. The patient has a favorable prognosis without recurrence. As far as we know, such a case has not been reported before. This report can increase the confidence in surgeries of mediastinal paragangliomas adhering tightly the adjacent structures.

## Author contributions

Conceptualization: Bing Zhang

Data curation: Bing Zhang, Guofang Liu

Methodology: Jian Li

Visualization: Bing Zhang, Guofang Liu

Writing-original draft: Bing Zhang

Writing-review & editing: Pinghua Wan
